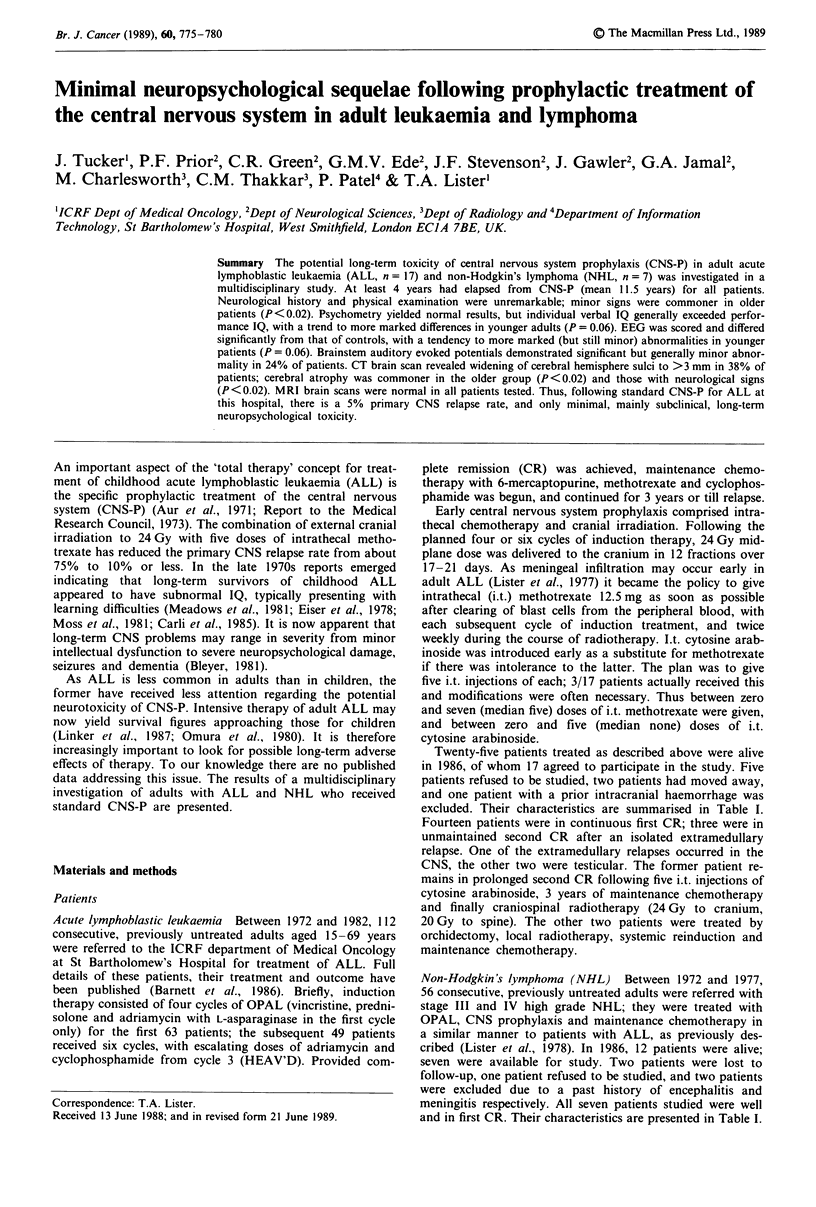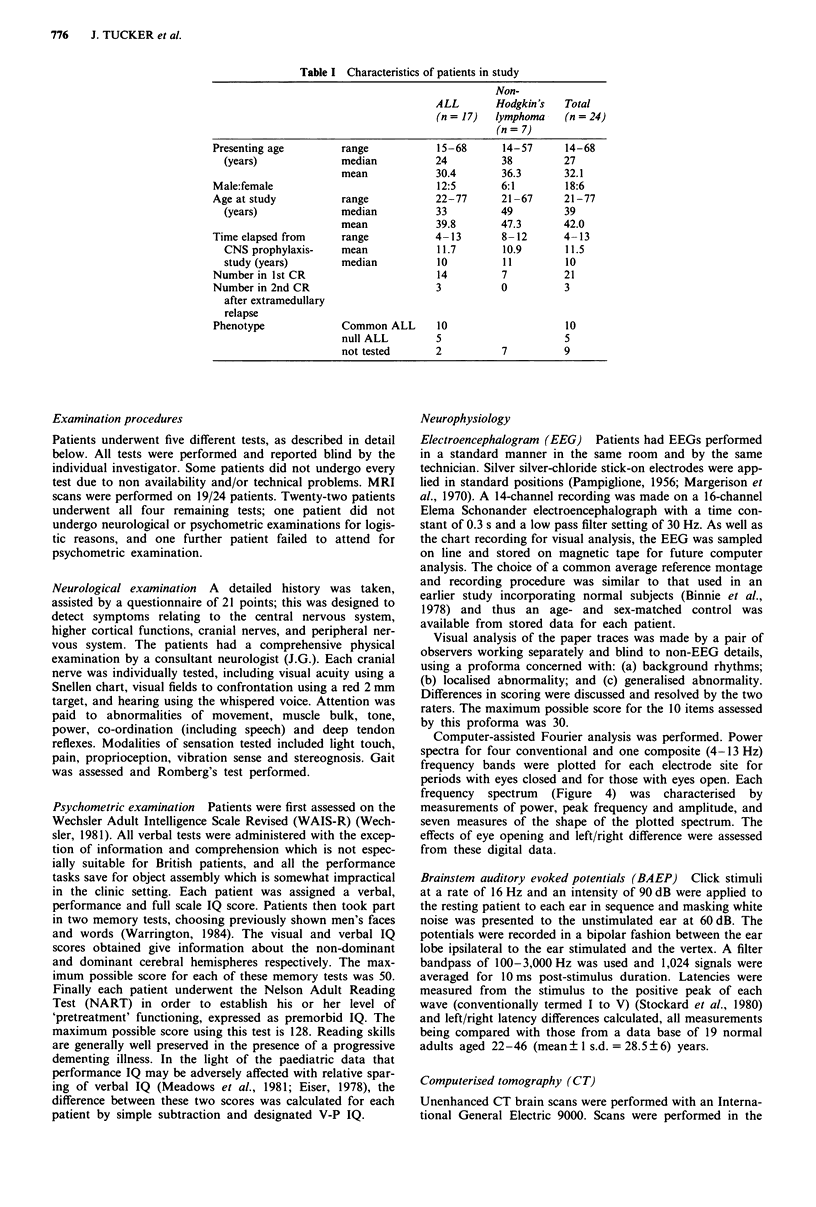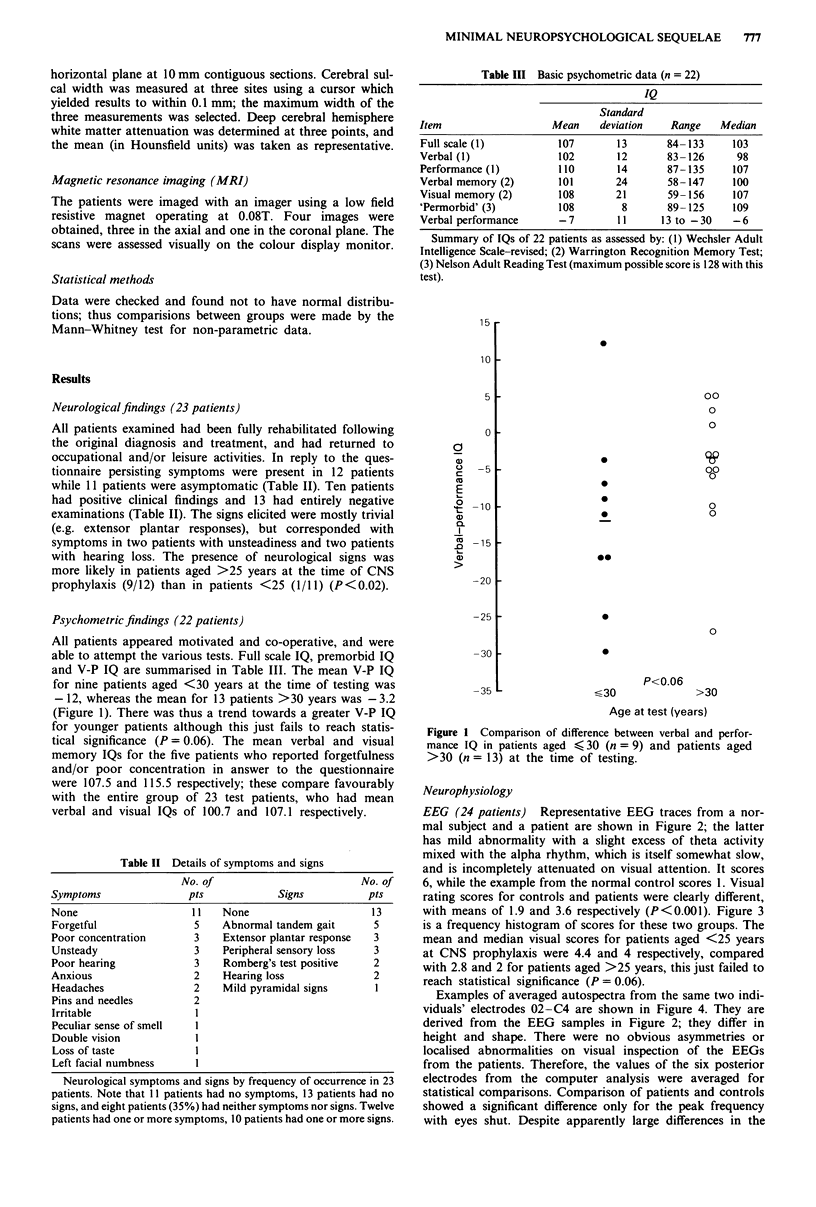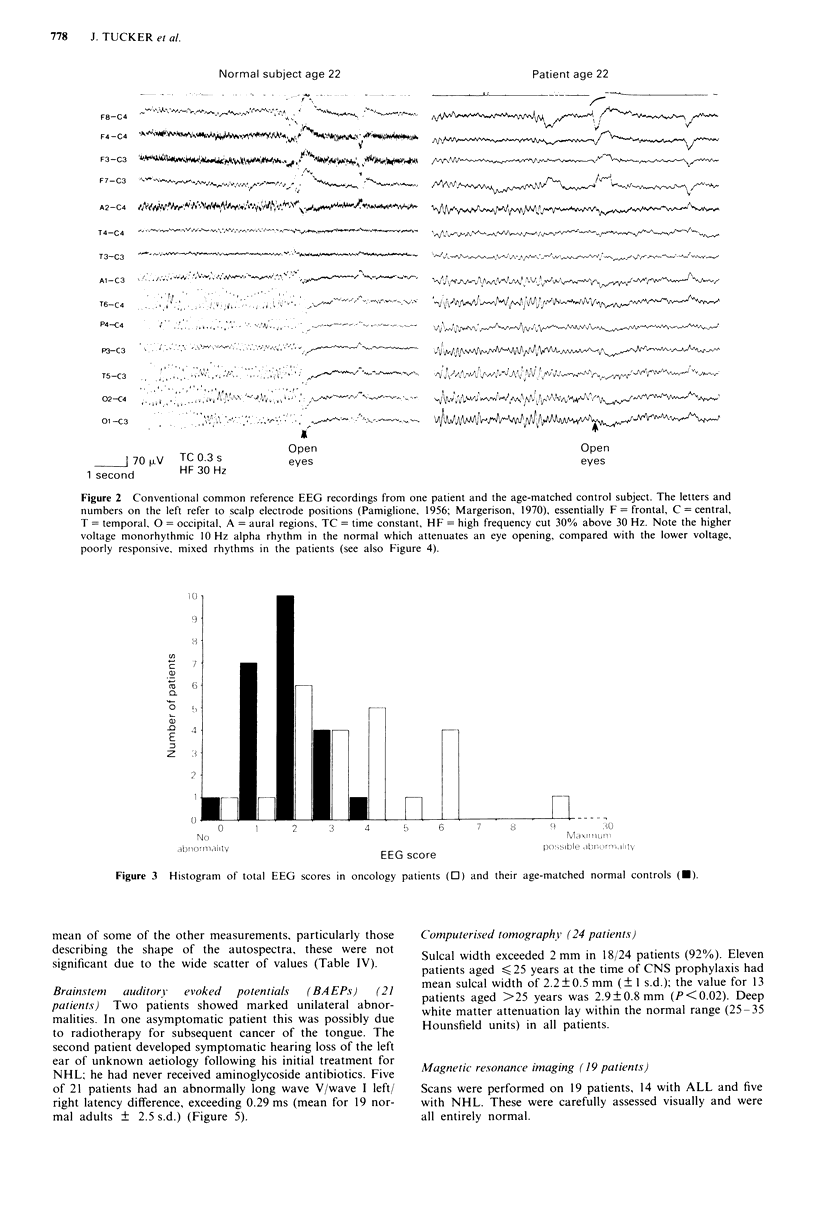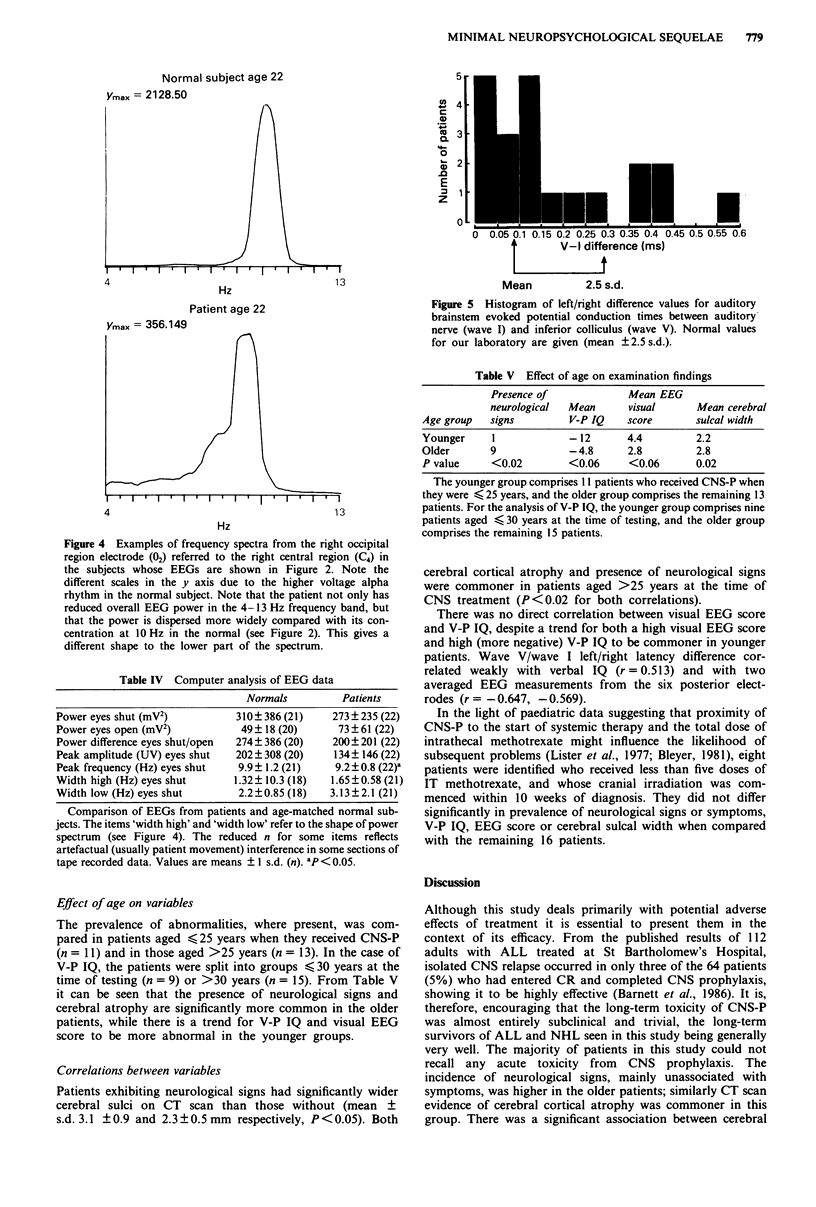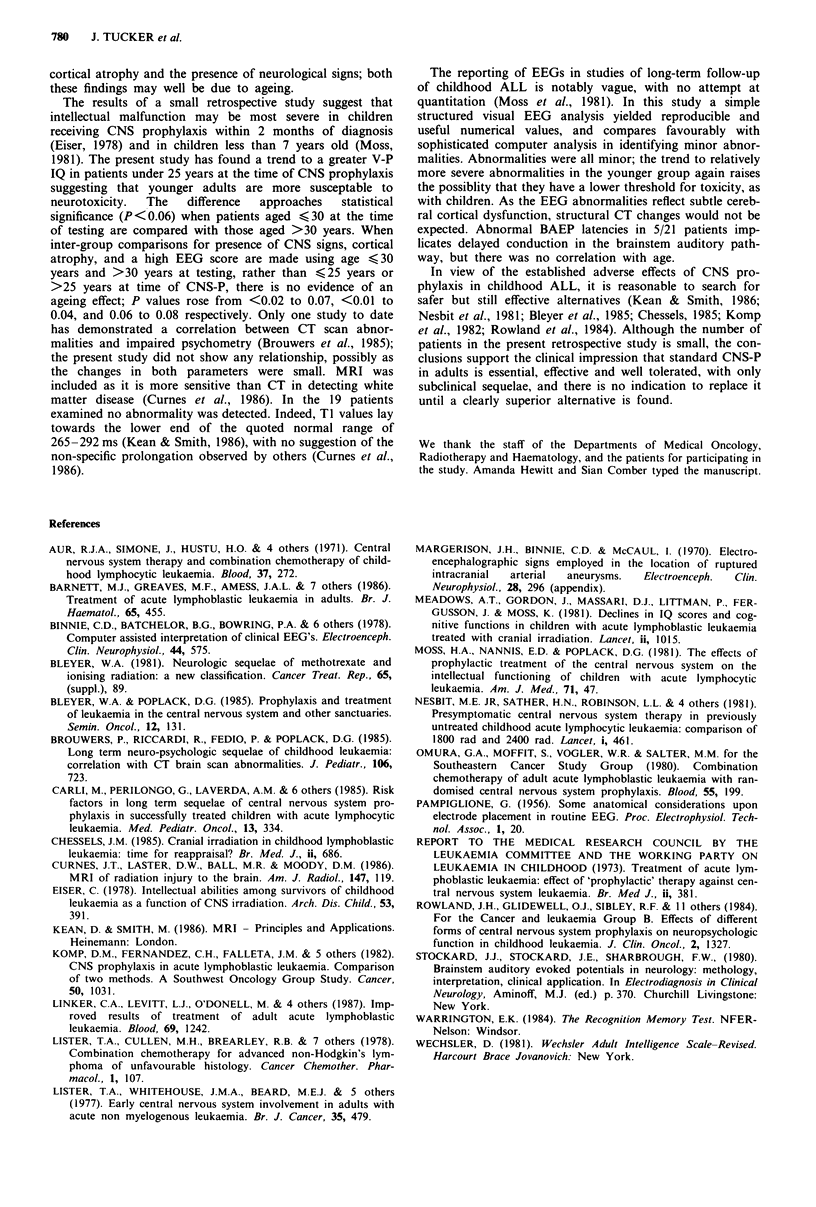# Minimal neuropsychological sequelae following prophylactic treatment of the central nervous system in adult leukaemia and lymphoma.

**DOI:** 10.1038/bjc.1989.358

**Published:** 1989-11

**Authors:** J. Tucker, P. F. Prior, C. R. Green, G. M. Ede, J. F. Stevenson, J. Gawler, G. A. Jamal, M. Charlesworth, C. M. Thakkar, P. Patel

**Affiliations:** ICRF Dept of Medical Oncology, St Bartholomew's Hospital, West Smithfield, London, UK.

## Abstract

The potential long-term toxicity of central nervous system prophylaxis (CNS-P) in adult acute lymphoblastic leukaemia (ALL, n = 17) and non-Hodgkin's lymphoma (NHL, n = 7) was investigated in a multidisciplinary study. At least 4 years had elapsed from CNS-P (mean 11.5 years) for all patients. Neurological history and physical examination were unremarkable; minor signs were commoner in older patients (P less than 0.02). Psychometry yielded normal results, but individual verbal IQ generally exceeded performance IQ, with a trend to more marked differences in younger adults (P = 0.06). EEG was scored and differed significantly from that of controls, with a tendency to more marked (but still minor) abnormalities in younger patients (P = 0.06). Brainstem auditory evoked potentials demonstrated significant but generally minor abnormality in 24% of patients. CT brain scan revealed widening of cerebral hemisphere sulci to greater than 3 mm in 38% of patients; cerebral atrophy was commoner in the older group (P less than 0.02) and those with neurological signs (P less than 0.02). MRI brain scans were normal in all patients tested. Thus, following standard CNS-P for ALL at this hospital, there is a 5% primary CNS relapse rate, and only minimal, mainly subclinical, long-term neuropsychological toxicity.


*Br. J. Cancer* (1989), **60**, 775–780 © The Macmillan Press Ltd., 1989

### Minimal neuropsychological sequelae following prophylactic treatment of the central nervous system in adult leukaemia and lymphoma

J. Tucker[1], P.F. Prior[2], C.R. Green[2], G.M.V. Ede[2], J.F. Stevenson[2], J. Gawler[2], G.A. Jamal[2], M. Charlesworth[3], C.M. Thakkar[3], P. Patel[4] & T.A. Lister[1]

[1]*ICRF Dept of Medical Oncology,* [2]*Dept of Neurological Sciences,* [3]*Dept of Radiology and* [4]*Department of Information Technology, St Bartholomew's Hospital, West Smithfield, London EC1A 7BE, UK.*

**Summary** The potential long-term toxicity of central nervous system prophylaxis (CNS-P) in adult acute lymphoblastic leukaemia (ALL, $n=17$) and non-Hodgkin's lymphoma (NHL, $n=7$) was investigated in a multidisciplinary study. At least 4 years had elapsed from CNS-P (mean 11.5 years) for all patients. Neurological history and physical examination were unremarkable; minor signs were commoner in older patients ($P<0.02$). Psychometry yielded normal results, but individual verbal IQ generally exceeded performance IQ, with a trend to more marked differences in younger adults ($P=0.06$). EEG was scored and differed significantly from that of controls, with a tendency to more marked (but still minor) abnormalities in younger patients ($P=0.06$). Brainstem auditory evoked potentials demonstrated significant but generally minor abnormality in 24% of patients. CT brain scan revealed widening of cerebral hemisphere sulci to >3 mm in 38% of patients; cerebral atrophy was commoner in the older group ($P<0.02$) and those with neurological signs ($P<0.02$). MRI brain scans were normal in all patients tested. Thus, following standard CNS-P for ALL at this hospital, there is a 5% primary CNS relapse rate, and only minimal, mainly subclinical, long-term neuropsychological toxicity.

An important aspect of the 'total therapy' concept for treatment of childhood acute lymphoblastic leukaemia (ALL) is the specific prophylactic treatment of the central nervous system (CNS-P) (Aur *et al.*, 1971; Report to the Medical Research Council, 1973). The combination of external cranial irradiation to 24 Gy with five doses of intrathecal methotrexate has reduced the primary CNS relapse rate from about 75% to 10% or less. In the late 1970s reports emerged indicating that long-term survivors of childhood ALL appeared to have subnormal IQ, typically presenting with learning difficulties (Meadows *et al.*, 1981; Eiser *et al.*, 1978; Moss *et al.*, 1981; Carli *et al.*, 1985). It is now apparent that long-term CNS problems may range in severity from minor intellectual dysfunction to severe neuropsychological damage, seizures and dementia (Bleyer, 1981).

As ALL is less common in adults than in children, the former have received less attention regarding the potential neurotoxicity of CNS-P. Intensive therapy of adult ALL may now yield survival figures approaching those for children (Linker *et al.*, 1987; Omura *et al.*, 1980). It is therefore increasingly important to look for possible long-term adverse effects of therapy. To our knowledge there are no published data addressing this issue. The results of a multidisciplinary investigation of adults with ALL and NHL who received standard CNS-P are presented.

### Materials and methods

### *Patients*

*Acute lymphoblastic leukaemia* Between 1972 and 1982, 112 consecutive, previously untreated adults aged 15–69 years were referred to the ICRF department of Medical Oncology at St Bartholomew's Hospital for treatment of ALL. Full details of these patients, their treatment and outcome have been published (Barnett *et al.*, 1986). Briefly, induction therapy consisted of four cycles of OPAL (vincristine, prednisolone and adriamycin with L-asparaginase in the first cycle only) for the first 63 patients; the subsequent 49 patients received six cycles, with escalating doses of adriamycin and cyclophosphamide from cycle 3 (HEAV'D). Provided complete remission (CR) was achieved, maintenance chemotherapy with 6-mercaptopurine, methotrexate and cyclophosphamide was begun, and continued for 3 years or till relapse.

Early central nervous system prophylaxis comprised intrathecal chemotherapy and cranial irradiation. Following the planned four or six cycles of induction therapy, 24 Gy midplane dose was delivered to the cranium in 12 fractions over 17–21 days. As meningeal infiltration may occur early in adult ALL (Lister *et al.*, 1977) it became the policy to give intrathecal (i.t.) methotrexate 12.5 mg as soon as possible after clearing of blast cells from the peripheral blood, with each subsequent cycle of induction treatment, and twice weekly during the course of radiotherapy. I.t. cytosine arabinoside was introduced early as a substitute for methotrexate if there was intolerance to the latter. The plan was to give five i.t. injections of each; 3/17 patients actually received this and modifications were often necessary. Thus between zero and seven (median five) doses of i.t. methotrexate were given, and between zero and five (median none) doses of i.t. cytosine arabinoside.

Twenty-five patients treated as described above were alive in 1986, of whom 17 agreed to participate in the study. Five patients refused to be studied, two patients had moved away, and one patient with a prior intracranial haemorrhage was excluded. Their characteristics are summarised in Table I. Fourteen patients were in continuous first CR; three were in unmaintained second CR after an isolated extramedullary relapse. One of the extramedullary relapses occurred in the CNS, the other two were testicular. The former patient remains in prolonged second CR following five i.t. injections of cytosine arabinoside, 3 years of maintenance chemotherapy and finally craniospinal radiotherapy (24 Gy to cranium, 20 Gy to spine). The other two patients were treated by orchidectomy, local radiotherapy, systemic reinduction and maintenance chemotherapy.

*Non-Hodgkin's lymphoma (NHL)* Between 1972 and 1977, 56 consecutive, previously untreated adults were referred with stage III and IV high grade NHL; they were treated with OPAL, CNS prophylaxis and maintenance chemotherapy in a similar manner to patients with ALL, as previously described (Lister *et al.*, 1978). In 1986, 12 patients were alive; seven were available for study. Two patients were lost to follow-up, one patient refused to be studied, and two patients were excluded due to a past history of encephalitis and meningitis respectively. All seven patients studied were well and in first CR. Their characteristics are presented in Table I.

Correspondence: T.A. Lister.
Received 13 June 1988; and in revised form 21 June 1989.

**Table I** Characteristics of patients in study

| | | *ALL (n = 17)* | *Non-Hodgkin's lymphoma (n = 7)* | *Total (n = 24)* |
|---|---|---|---|---|
| Presenting age (years) | range | 15–68 | 14–57 | 14–68 |
| | median | 24 | 38 | 27 |
| | mean | 30.4 | 36.3 | 32.1 |
| Male:female | | 12:5 | 6:1 | 18:6 |
| Age at study (years) | range | 22–77 | 21–67 | 21–77 |
| | median | 33 | 49 | 39 |
| | mean | 39.8 | 47.3 | 42.0 |
| Time elapsed from CNS prophylaxis-study (years) | range | 4–13 | 8–12 | 4–13 |
| | mean | 11.7 | 10.9 | 11.5 |
| | median | 10 | 11 | 10 |
| Number in 1st CR | | 14 | 7 | 21 |
| Number in 2nd CR after extramedullary relapse | | 3 | 0 | 3 |
| Phenotype | Common ALL | 10 | | 10 |
| | null ALL | 5 | | 5 |
| | not tested | 2 | 7 | 9 |

*Examination procedures*

Patients underwent five different tests, as described in detail below. All tests were performed and reported blind by the individual investigator. Some patients did not undergo every test due to non availability and/or technical problems. MRI scans were performed on 19/24 patients. Twenty-two patients underwent all four remaining tests; one patient did not undergo neurological or psychometric examinations for logistic reasons, and one further patient failed to attend for psychometric examination.

*Neurological examination* A detailed history was taken, assisted by a questionnaire of 21 points; this was designed to detect symptoms relating to the central nervous system, higher cortical functions, cranial nerves, and peripheral nervous system. The patients had a comprehensive physical examination by a consultant neurologist (J.G.). Each cranial nerve was individually tested, including visual acuity using a Snellen chart, visual fields to confrontation using a red 2 mm target, and hearing using the whispered voice. Attention was paid to abnormalities of movement, muscle bulk, tone, power, co-ordination (including speech) and deep tendon reflexes. Modalities of sensation tested included light touch, pain, proprioception, vibration sense and stereognosis. Gait was assessed and Romberg's test performed.

*Psychometric examination* Patients were first assessed on the Wechsler Adult Intelligence Scale Revised (WAIS-R) (Wechsler, 1981). All verbal tests were administered with the exception of information and comprehension which is not especially suitable for British patients, and all the performance tasks save for object assembly which is somewhat impractical in the clinic setting. Each patient was assigned a verbal, performance and full scale IQ score. Patients then took part in two memory tests, choosing previously shown men's faces and words (Warrington, 1984). The visual and verbal IQ scores obtained give information about the non-dominant and dominant cerebral hemispheres respectively. The maximum possible score for each of these memory tests was 50. Finally each patient underwent the Nelson Adult Reading Test (NART) in order to establish his or her level of 'pretreatment' functioning, expressed as premorbid IQ. The maximum possible score using this test is 128. Reading skills are generally well preserved in the presence of a progressive dementing illness. In the light of the paediatric data that performance IQ may be adversely affected with relative sparing of verbal IQ (Meadows et al., 1981; Eiser, 1978), the difference between these two scores was calculated for each patient by simple subtraction and designated V-P IQ.

*Neurophysiology*

*Electroencephalogram (EEG)* Patients had EEGs performed in a standard manner in the same room and by the same technician. Silver silver-chloride stick-on electrodes were applied in standard positions (Pampiglione, 1956; Margerison et al., 1970). A 14-channel recording was made on a 16-channel Elema Schonander electroencephalograph with a time constant of 0.3 s and a low pass filter setting of 30 Hz. As well as the chart recording for visual analysis, the EEG was sampled on line and stored on magnetic tape for future computer analysis. The choice of a common average reference montage and recording procedure was similar to that used in an earlier study incorporating normal subjects (Binnie et al., 1978) and thus an age- and sex-matched control was available from stored data for each patient.

Visual analysis of the paper traces was made by a pair of observers working separately and blind to non-EEG details, using a proforma concerned with: (a) background rhythms; (b) localised abnormality; and (c) generalised abnormality. Differences in scoring were discussed and resolved by the two raters. The maximum possible score for the 10 items assessed by this proforma was 30.

Computer-assisted Fourier analysis was performed. Power spectra for four conventional and one composite (4–13 Hz) frequency bands were plotted for each electrode site for periods with eyes closed and for those with eyes open. Each frequency spectrum (Figure 4) was characterised by measurements of power, peak frequency and amplitude, and seven measures of the shape of the plotted spectrum. The effects of eye opening and left/right difference were assessed from these digital data.

*Brainstem auditory evoked potentials (BAEP)* Click stimuli at a rate of 16 Hz and an intensity of 90 dB were applied to the resting patient to each ear in sequence and masking white noise was presented to the unstimulated ear at 60 dB. The potentials were recorded in a bipolar fashion between the ear lobe ipsilateral to the ear stimulated and the vertex. A filter bandpass of 100–3,000 Hz was used and 1,024 signals were averaged for 10 ms post-stimulus duration. Latencies were measured from the stimulus to the positive peak of each wave (conventionally termed I to V) (Stockard et al., 1980) and left/right latency differences calculated, all measurements being compared with those from a data base of 19 normal adults aged 22–46 (mean $\pm$ 1 s.d. = 28.5 $\pm$ 6) years.

*Computerised tomography (CT)*

Unenhanced CT brain scans were performed with an International General Electric 9000. Scans were performed in the horizontal plane at 10 mm contiguous sections. Cerebral sulcal width was measured at three sites using a cursor which yielded results to within 0.1 mm; the maximum width of the three measurements was selected. Deep cerebral hemisphere white matter attenuation was determined at three points, and the mean (in Hounsfield units) was taken as representative.

*Magnetic resonance imaging (MRI)*

The patients were imaged with an imager using a low field resistive magnet operating at 0.08T. Four images were obtained, three in the axial and one in the coronal plane. The scans were assessed visually on the colour display monitor.

*Statistical methods*

Data were checked and found not to have normal distributions; thus comparisions between groups were made by the Mann–Whitney test for non-parametric data.

### Results

*Neurological findings (23 patients)*

All patients examined had been fully rehabilitated following the original diagnosis and treatment, and had returned to occupational and/or leisure activities. In reply to the questionnaire persisting symptoms were present in 12 patients while 11 patients were asymptomatic (Table II). Ten patients had positive clinical findings and 13 had entirely negative examinations (Table II). The signs elicited were mostly trivial (e.g. extensor plantar responses), but corresponded with symptoms in two patients with unsteadiness and two patients with hearing loss. The presence of neurological signs was more likely in patients aged >25 years at the time of CNS prophylaxis (9/12) than in patients <25 (1/11) ($P<0.02$).

*Psychometric findings (22 patients)*

All patients appeared motivated and co-operative, and were able to attempt the various tests. Full scale IQ, premorbid IQ and V-P IQ are summarised in Table III. The mean V-P IQ for nine patients aged <30 years at the time of testing was −12, whereas the mean for 13 patients >30 years was −3.2 (Figure 1). There was thus a trend towards a greater V-P IQ for younger patients although this just fails to reach statistical significance ($P=0.06$). The mean verbal and visual memory IQs for the five patients who reported forgetfulness and/or poor concentration in answer to the questionnaire were 107.5 and 115.5 respectively; these compare favourably with the entire group of 23 test patients, who had mean verbal and visual IQs of 100.7 and 107.1 respectively.

**Table II** Details of symptoms and signs

| *Symptoms* | *No. of pts* | *Signs* | *No. of pts* |
|---|---|---|---|
| None | 11 | None | 13 |
| Forgetful | 5 | Abnormal tandem gait | 5 |
| Poor concentration | 3 | Extensor plantar response | 3 |
| Unsteady | 3 | Peripheral sensory loss | 3 |
| Poor hearing | 3 | Romberg's test positive | 2 |
| Anxious | 2 | Hearing loss | 2 |
| Headaches | 2 | Mild pyramidal signs | 1 |
| Pins and needles | 2 | | |
| Irritable | 1 | | |
| Peculiar sense of smell | 1 | | |
| Double vision | 1 | | |
| Loss of taste | 1 | | |
| Left facial numbness | 1 | | |

Neurological symptoms and signs by frequency of occurrence in 23 patients. Note that 11 patients had no symptoms, 13 patients had no signs, and eight patients (35%) had neither symptoms nor signs. Twelve patients had one or more symptoms, 10 patients had one or more signs.

**Table III** Basic psychometric data ($n=22$)

| *Item* | *IQ* *Mean* | *Standard deviation* | *Range* | *Median* |
|---|---|---|---|---|
| Full scale (1) | 107 | 13 | 84–133 | 103 |
| Verbal (1) | 102 | 12 | 83–126 | 98 |
| Performance (1) | 110 | 14 | 87–135 | 107 |
| Verbal memory (2) | 101 | 24 | 58–147 | 100 |
| Visual memory (2) | 108 | 21 | 59–156 | 107 |
| 'Permorbid' (3) | 108 | 8 | 89–125 | 109 |
| Verbal performance | −7 | 11 | 13 to −30 | −6 |

Summary of IQs of 22 patients as assessed by: (1) Wechsler Adult Intelligence Scale–revised; (2) Warrington Recognition Memory Test; (3) Nelson Adult Reading Test (maximum possible score is 128 with this test).

**Figure 1** Comparison of difference between verbal and performance IQ in patients aged ≤30 ($n=9$) and patients aged >30 ($n=13$) at the time of testing.

*Neurophysiology*

*EEG (24 patients)* Representative EEG traces from a normal subject and a patient are shown in Figure 2; the latter has mild abnormality with a slight excess of theta activity mixed with the alpha rhythm, which is itself somewhat slow, and is incompletely attenuated on visual attention. It scores 6, while the example from the normal control scores 1. Visual rating scores for controls and patients were clearly different, with means of 1.9 and 3.6 respectively ($P<0.001$). Figure 3 is a frequency histogram of scores for these two groups. The mean and median visual scores for patients aged <25 years at CNS prophylaxis were 4.4 and 4 respectively, compared with 2.8 and 2 for patients aged >25 years, this just failed to reach statistical significance ($P=0.06$).

Examples of averaged autospectra from the same two individuals' electrodes 02–C4 are shown in Figure 4. They are derived from the EEG samples in Figure 2; they differ in height and shape. There were no obvious asymmetries or localised abnormalities on visual inspection of the EEGs from the patients. Therefore, the values of the six posterior electrodes from the computer analysis were averaged for statistical comparisons. Comparison of patients and controls showed a significant difference only for the peak frequency with eyes shut. Despite apparently large differences in the

**Figure 2** Conventional common reference EEG recordings from one patient and the age-matched control subject. The letters and numbers on the left refer to scalp electrode positions (Pamiglione, 1956; Margerison, 1970), essentially F = frontal, C = central, T = temporal, O = occipital, A = aural regions, TC = time constant, HF = high frequency cut 30% above 30 Hz. Note the higher voltage monorhythmic 10 Hz alpha rhythm in the normal which attenuates an eye opening, compared with the lower voltage, poorly responsive, mixed rhythms in the patients (see also Figure 4).

**Figure 3** Histogram of total EEG scores in oncology patients (□) and their age-matched normal controls (■).

mean of some of the other measurements, particularly those describing the shape of the autospectra, these were not significant due to the wide scatter of values (Table IV).

*Brainstem auditory evoked potentials (BAEPs) (21 patients)* Two patients showed marked unilateral abnormalities. In one asymptomatic patient this was possibly due to radiotherapy for subsequent cancer of the tongue. The second patient developed symptomatic hearing loss of the left ear of unknown aetiology following his initial treatment for NHL; he had never received aminoglycoside antibiotics. Five of 21 patients had an abnormally long wave V/wave I left/right latency difference, exceeding 0.29 ms (mean for 19 normal adults $\pm$ 2.5 s.d.) (Figure 5).

*Computerised tomography (24 patients)*

Sulcal width exceeded 2 mm in 18/24 patients (92%). Eleven patients aged $\leqslant$25 years at the time of CNS prophylaxis had mean sulcal width of 2.2$\pm$0.5 mm ($\pm$1 s.d.); the value for 13 patients aged $>$25 years was 2.9$\pm$0.8 mm ($P<0.02$). Deep white matter attenuation lay within the normal range (25–35 Hounsfield units) in all patients.

*Magnetic resonance imaging (19 patients)*

Scans were performed on 19 patients, 14 with ALL and five with NHL. These were carefully assessed visually and were all entirely normal.

**Figure 4** Examples of frequency spectra from the right occipital region electrode ($0_2$) referred to the right central region ($C_4$) in the subjects whose EEGs are shown in Figure 2. Note the different scales in the *y* axis due to the higher voltage alpha rhythm in the normal subject. Note that the patient not only has reduced overall EEG power in the 4–13 Hz frequency band, but that the power is dispersed more widely compared with its concentration at 10 Hz in the normal (see Figure 2). This gives a different shape to the lower part of the spectrum.

**Table IV** Computer analysis of EEG data

| | *Normals* | *Patients* |
|---|---|---|
| Power eyes shut ($mV^2$) | 310±386 (21) | 273±235 (22) |
| Power eyes open ($mV^2$) | 49±18 (20) | 73±61 (22) |
| Power difference eyes shut/open | 274±386 (20) | 200±201 (22) |
| Peak amplitude (UV) eyes shut | 202±308 (20) | 134±146 (22) |
| Peak frequency (Hz) eyes shut | 9.9±1.2 (21) | 9.2±0.8 (22)[a] |
| Width high (Hz) eyes shut | 1.32±10.3 (18) | 1.65±0.58 (21) |
| Width low (Hz) eyes shut | 2.2±0.85 (18) | 3.13±2.1 (21) |

Comparison of EEGs from patients and age-matched normal subjects. The items 'width high' and 'width low' refer to the shape of power spectrum (see Figure 4). The reduced *n* for some items reflects artefactual (usually patient movement) interference in some sections of tape recorded data. Values are means ±1 s.d. (*n*). [a]$P<0.05$.

### *Effect of age on variables*

The prevalence of abnormalities, where present, was compared in patients aged ≤25 years when they received CNS-P ($n = 11$) and in those aged >25 years ($n = 13$). In the case of V-P IQ, the patients were split into groups ≤30 years at the time of testing ($n = 9$) or >30 years ($n = 15$). From Table V it can be seen that the presence of neurological signs and cerebral atrophy are significantly more common in the older patients, while there is a trend for V-P IQ and visual EEG score to be more abnormal in the younger groups.

### *Correlations between variables*

Patients exhibiting neurological signs had significantly wider cerebral sulci on CT scan than those without (mean ± s.d. 3.1 ±0.9 and 2.3±0.5 mm respectively, $P<0.05$). Both

**Figure 5** Histogram of left/right difference values for auditory brainstem evoked potential conduction times between auditory nerve (wave I) and inferior colliculus (wave V). Normal values for our laboratory are given (mean ±2.5 s.d.).

**Table V** Effect of age on examination findings

| *Age group* | *Presence of neurological signs* | *Mean V-P IQ* | *Mean EEG visual score* | *Mean cerebral sulcal width* |
|---|---|---|---|---|
| Younger | 1 | −12 | 4.4 | 2.2 |
| Older | 9 | −4.8 | 2.8 | 2.8 |
| *P* value | <0.02 | <0.06 | <0.06 | 0.02 |

The younger group comprises 11 patients who received CNS-P when they were ≤25 years, and the older group comprises the remaining 13 patients. For the analysis of V-P IQ, the younger group comprises nine patients aged ≤30 years at the time of testing, and the older group comprises the remaining 15 patients.

cerebral cortical atrophy and presence of neurological signs were commoner in patients aged >25 years at the time of CNS treatment ($P<0.02$ for both correlations).

There was no direct correlation between visual EEG score and V-P IQ, despite a trend for both a high visual EEG score and high (more negative) V-P IQ to be commoner in younger patients. Wave V/wave I left/right latency difference correlated weakly with verbal IQ ($r = 0.513$) and with two averaged EEG measurements from the six posterior electrodes ($r = -0.647$, $-0.569$).

In the light of paediatric data suggesting that proximity of CNS-P to the start of systemic therapy and the total dose of intrathecal methotrexate might influence the likelihood of subsequent problems (Lister *et al.*, 1977; Bleyer, 1981), eight patients were identified who received less than five doses of IT methotrexate, and whose cranial irradiation was commenced within 10 weeks of diagnosis. They did not differ significantly in prevalence of neurological signs or symptoms, V-P IQ, EEG score or cerebral sulcal width when compared with the remaining 16 patients.

### Discussion

Although this study deals primarily with potential adverse effects of treatment it is essential to present them in the context of its efficacy. From the published results of 112 adults with ALL treated at St Bartholomew's Hospital, isolated CNS relapse occurred in only three of the 64 patients (5%) who had entered CR and completed CNS prophylaxis, showing it to be highly effective (Barnett *et al.*, 1986). It is, therefore, encouraging that the long-term toxicity of CNS-P was almost entirely subclinical and trivial, the long-term survivors of ALL and NHL seen in this study being generally very well. The majority of patients in this study could not recall any acute toxicity from CNS prophylaxis. The incidence of neurological signs, mainly unassociated with symptoms, was higher in the older patients; similarly CT scan evidence of cerebral cortical atrophy was commoner in this group. There was a significant association between cerebral cortical atrophy and the presence of neurological signs; both these findings may well be due to ageing.

The results of a small retrospective study suggest that intellectual malfunction may be most severe in children receiving CNS prophylaxis within 2 months of diagnosis (Eiser, 1978) and in children less than 7 years old (Moss, 1981). The present study has found a trend to a greater V-P IQ in patients under 25 years at the time of CNS prophylaxis suggesting that younger adults are more susceptable to neurotoxicity. The difference approaches statistical significance ($P<0.06$) when patients aged $\leqslant 30$ at the time of testing are compared with those aged $>30$ years. When inter-group comparisons for presence of CNS signs, cortical atrophy, and a high EEG score are made using age $\leqslant 30$ years and $>30$ years at testing, rather than $\leqslant 25$ years or $>25$ years at time of CNS-P, there is no evidence of an ageing effect; $P$ values rose from $<0.02$ to 0.07, $<0.01$ to 0.04, and 0.06 to 0.08 respectively. Only one study to date has demonstrated a correlation between CT scan abnormalities and impaired psychometry (Brouwers *et al.*, 1985); the present study did not show any relationship, possibly as the changes in both parameters were small. MRI was included as it is more sensitive than CT in detecting white matter disease (Curnes *et al.*, 1986). In the 19 patients examined no abnormality was detected. Indeed, T1 values lay towards the lower end of the quoted normal range of 265–292 ms (Kean & Smith, 1986), with no suggestion of the non-specific prolongation observed by others (Curnes *et al.*, 1986).

The reporting of EEGs in studies of long-term follow-up of childhood ALL is notably vague, with no attempt at quantitation (Moss *et al.*, 1981). In this study a simple structured visual EEG analysis yielded reproducible and useful numerical values, and compares favourably with sophisticated computer analysis in identifying minor abnormalities. Abnormalities were all minor; the trend to relatively more severe abnormalities in the younger group again raises the possiblity that they have a lower threshold for toxicity, as with children. As the EEG abnormalities reflect subtle cerebral cortical dysfunction, structural CT changes would not be expected. Abnormal BAEP latencies in 5/21 patients implicates delayed conduction in the brainstem auditory pathway, but there was no correlation with age.

In view of the established adverse effects of CNS prophylaxis in childhood ALL, it is reasonable to search for safer but still effective alternatives (Kean & Smith, 1986; Nesbit *et al.*, 1981; Bleyer *et al.*, 1985; Chessels, 1985; Komp *et al.*, 1982; Rowland *et al.*, 1984). Although the number of patients in the present retrospective study is small, the conclusions support the clinical impression that standard CNS-P in adults is essential, effective and well tolerated, with only subclinical sequelae, and there is no indication to replace it until a clearly superior alternative is found.

We thank the staff of the Departments of Medical Oncology, Radiotherapy and Haematology, and the patients for participating in the study. Amanda Hewitt and Sian Comber typed the manuscript.

### References

AUR, R.J.A., SIMONE, J., HUSTU, H.O. & 4 others (1971). Central nervous system therapy and combination chemotherapy of childhood lymphocytic leukaemia. *Blood*, **37**, 272.

BARNETT, M.J., GREAVES, M.F., AMESS, J.A.L. & 7 others (1986). Treatment of acute lymphoblastic leukaemia in adults. *Br. J. Haematol.*, **65**, 455.

BINNIE, C.D., BATCHELOR, B.G., BOWRING, P.A. & 6 others (1978). Computer assisted interpretation of clinical EEG's. *Electroenceph. Clin. Neurophysiol.*, **44**, 575.

BLEYER, W.A. (1981). Neurologic sequelae of methotrexate and ionising radiation: a new classification. *Cancer Treat. Rep.*, **65**, (suppl.), 89.

BLEYER, W.A. & POPLACK, D.G. (1985). Prophylaxis and treatment of leukaemia in the central nervous system and other sanctuaries. *Semin. Oncol.*, **12**, 131.

BROUWERS, P., RICCARDI, R., FEDIO, P. & POPLACK, D.G. (1985). Long term neuro-psychologic sequelae of childhood leukaemia: correlation with CT brain scan abnormalities. *J. Pediatr.*, **106**, 723.

CARLI, M., PERILONGO, G., LAVERDA, A.M. & 6 others (1985). Risk factors in long term sequelae of central nervous system prophylaxis in successfully treated children with acute lymphocytic leukaemia. *Med. Pediatr. Oncol.*, **13**, 334.

CHESSELS, J.M. (1985). Cranial irradiation in childhood lymphoblastic leukaemia: time for reappraisal? *Br. Med. J.*, **ii**, 686.

CURNES, J.T., LASTER, D.W., BALL, M.R. & MOODY, D.M. (1986). MRI of radiation injury to the brain. *Am. J. Radiol.*, **147**, 119.

EISER, C. (1978). Intellectual abilities among survivors of childhood leukaemia as a function of CNS irradiation. *Arch. Dis. Child.*, **53**, 391.

KEAN, D. & SMITH, M. (1986). MRI – Principles and Applications. Heinemann: London.

KOMP, D.M., FERNANDEZ, C.H., FALLETA, J.M. & 5 others (1982). CNS prophylaxis in acute lymphoblastic leukaemia. Comparison of two methods. A Southwest Oncology Group Study. *Cancer*, **50**, 1031.

LINKER, C.A., LEVITT, L.J., O'DONELL, M. & 4 others (1987). Improved results of treatment of adult acute lymphoblastic leukaemia. *Blood*, **69**, 1242.

LISTER, T.A., CULLEN, M.H., BREARLEY, R.B. & 7 others (1978). Combination chemotherapy for advanced non-Hodgkin's lymphoma of unfavourable histology. *Cancer Chemother. Pharmacol.*, **1**, 107.

LISTER, T.A., WHITEHOUSE, J.M.A., BEARD, M.E.J. & 5 others (1977). Early central nervous system involvement in adults with acute non myelogenous leukaemia. *Br. J. Cancer*, **35**, 479.

MARGERISON, J.H., BINNIE, C.D. & MCCAUL, I. (1970). Electroencephalographic signs employed in the location of ruptured intracranial arterial aneurysms. *Electroenceph. Clin. Neurophysiol.*, **28**, 296 (appendix).

MEADOWS, A.T., GORDON, J., MASSARI, D.J., LITTMAN, P., FERGUSSON, J. & MOSS, K. (1981). Declines in IQ scores and cognitive functions in children with acute lymphoblastic leukaemia treated with cranial irradiation. *Lancet*, **ii**, 1015.

MOSS, H.A., NANNIS, E.D. & POPLACK, D.G. (1981). The effects of prophylactic treatment of the central nervous system on the intellectual functioning of children with acute lymphocytic leukaemia. *Am. J. Med.*, **71**, 47.

NESBIT, M.E. JR, SATHER, H.N., ROBINSON, L.L. & 4 others (1981). Presymptomatic central nervous system therapy in previously untreated childhood acute lymphocytic leukaemia: comparison of 1800 rad and 2400 rad. *Lancet*, **i**, 461.

OMURA, G.A., MOFFIT, S., VOGLER, W.R. & SALTER, M.M. for the Southeastern Cancer Study Group (1980). Combination chemotherapy of adult acute lymphoblastic leukaemia with randomised central nervous system prophylaxis. *Blood*, **55**, 199.

PAMPIGLIONE, G. (1956). Some anatomical considerations upon electrode placement in routine EEG. *Proc. Electrophysiol. Technol. Assoc.*, **1**, 20.

REPORT TO THE MEDICAL RESEARCH COUNCIL BY THE LEUKAEMIA COMMITTEE AND THE WORKING PARTY ON LEUKAEMIA IN CHILDHOOD (1973). Treatment of acute lymphoblastic leukaemia: effect of 'prophylactic' therapy against central nervous system leukaemia. *Br. Med J.*, **ii**, 381.

ROWLAND, J.H., GLIDEWELL, O.J., SIBLEY, R.F. & 11 others (1984). For the Cancer and leukaemia Group B. Effects of different forms of central nervous system prophylaxis on neuropsychologic function in childhood leukaemia. *J. Clin. Oncol.*, **2**, 1327.

STOCKARD, J.J., STOCKARD, J.E., SHARBROUGH, F.W., (1980). Brainstem auditory evoked potentials in neurology: methology, interpretation, clinical application. In *Electrodiagnosis in Clinical Neurology*, Aminoff, M.J. (ed.) p. 370. Churchill Livingstone: New York.

WARRINGTON, E.K. (1984). *The Recognition Memory Test*. NFER-Nelson: Windsor.

WECHSLER, D. (1981). *Wechsler Adult Intelligence Scale-Revised. Harcourt Brace Jovanovich:* New York.